# Spatial Patterns of the Indications of Acupoints Using Data Mining in Classic Medical Text: A Possible Visualization of the Meridian System

**DOI:** 10.1155/2015/457071

**Published:** 2015-10-11

**Authors:** Won-Mo Jung, Taehyung Lee, In-Seon Lee, Sanghyun Kim, Hyunchul Jang, Song-Yi Kim, Hi-Joon Park, Younbyoung Chae

**Affiliations:** ^1^Acupuncture and Meridian Science Research Center, Kyung Hee University, Seoul 02447, Republic of Korea; ^2^Department of Medical History, College of Korean Medicine, Kyung Hee University, Seoul 02447, Republic of Korea; ^3^Mibyeong Research Center, Korea Institute of Oriental Medicine, Daejeon 34054, Republic of Korea

## Abstract

The indications of acupoints are thought to be highly associated with the lines of the meridian systems. The present study used data mining methods to analyze the characteristics of the indications of each acupoint and to visualize the relationships between the acupoints and disease sites in the classic Korean medical text *Chimgoogyeongheombang*. Using a term frequency-inverse document frequency (tf-idf) scheme, the present study extracted valuable data regarding the indications of each acupoint according to the frequency of the cooccurrences of eight Source points and eighteen disease sites. Furthermore, the spatial patterns of the indications of each acupoint on a body map were visualized according to the tf-idf values. Each acupoint along the different meridians exhibited different constellation patterns at various disease sites. Additionally, the spatial patterns of the indications of each acupoint were highly associated with the route of the corresponding meridian. The present findings demonstrate that the indications of each acupoint were primarily associated with the corresponding meridian system. Furthermore, these findings suggest that the routes of the meridians may have clinical implications in terms of identifying the constellations of the indications of acupoints.

## 1. Introduction

In many instances, one diagram is better than a thousand words. For example, a famous physician from the early stages of the Tang Dynasty, Sun Si-miao, declared that an acupoint could not be well located without graphic guidance. The ancient Chinese invented several methods for displaying information about acupoints and meridians on the human body surface by relying on ancient infographics of the meridian system rather than a detailed knowledge of human anatomy. Additionally, they described empirical clinical information in terms of the selection of appropriate acupoints for the treatment of specific diseases. However, when Western people were exposed to the Chinese acupuncture map in the late 17th century, it was necessary for them to understand the acupuncture point system using anatomical knowledge. As a result, to comply with the contemporary conventions of European anatomical atlases, the first versions of the Chinese acupuncture map for the Western world were embellished with dissected flaps of skin at the head [1]. From a historical point of view, the Eastern texts* Mingtang Diagram*,* Diagram of Meridian and Collaterals*, and* Bronze Statue* were also gradually influenced by Western-style anatomy. However, despite decades of research and a merging of Western and Eastern ideals, an anatomical map of the meridians on the human body has yet to be fully realized [2]. It has been suggested that high electrical conductance, acupuncture sensation patterns, and possible relationships with connective tissue planes could represent meridians or act as identifiers for meridians [3–6]. However, none of these studies can fully explain the relationships that exist between treatment at each acupoint and the subsequent clinical improvements. Thus, one must understand the origin and clinical significance of the meridian system in order to fully understand the acupuncture process that is based on this system.

Traditional East Asian medical techniques can be used to diagnose diseases of visceral organs, such as the stomach or kidneys, by the simple application of the appropriate palpations on the arteries around particular body sites. There are intriguing similarities between Western medicine and traditional bloodletting sites and acupoints, but Hippocratic treatments using venesection have largely lost their topographical importance and disappeared from the Western medicine [7]. In traditional East Asian medicine, on the other hand, the relationships among acupoints and disease sites are generally understood based on an empirical knowledge of the meridian system which functions as the underlying template for acupuncture treatment [8, 9]. In and of itself, the meridian system is also considered to be a diagnostic tool useful for the identification of diseases or symptoms along the meridian lines and for the association of the pathology of diseases or symptoms with the relevant organ [10, 11]. The route of a meridian system exemplifies the constellations of the indications of acupoints. In other words, the indications of acupoints have a high association with the lines of the meridian systems and, in this manner, the acupoints may be connected with a suspected disease or symptom to form a network of knowledge that is the meridian system [12, 13]. As a theoretical model of the indications of acupoints, the meridian system can further the current understanding of the interconnections that underlie the pathologies of particular diseases or symptoms [14]. Moreover, based on an ever-increasing amount of empirical clinical data regarding the indications of acupoints, the meridian system has been continually amended and developed in association with advances in medicine.

Given that the meridian system proposes that there are a series of connections among different areas, organs, and functions, this system may be visualized using the indications of acupoints. Whenever practitioners treat patients using acupuncture and moxibustion, the appropriate selection of acupoints is made based on three fundamental principles: (1) local acupoints near the area where the symptoms are occurring, (2) distant acupoints along the meridian, and (3) distant acupoints based on symptom differentiation [14, 15]. For example, when patients are suffering from a toothache and facial edema, the practitioner can consider diseases associated with the Large Intestine meridian and choose local acupoints such as LI 19 and LI 20 and distant acupoints such as LI 4 and LI 5. To clarify the unique characteristics of traditional East Asian medicinal techniques, several studies have used data mining and network science to characterize the relationships that exist among the symptoms of patients and East Asian treatment methods [14, 16–19]. In recent years, it has become the norm for acupuncture practitioners to develop a practical clinical decision prior to treatment using the modern methodologies of evidence-based medicine and the abundant information readily available in current scientific databases [20]. Additionally, the field of acupuncture has used data mining algorithms to uncover useful patterns of symptoms and treatment methods within various fields of research, including those from the classic texts of traditional East Asian medicine [21, 22].

Every disease or symptom can be treated by a wide assortment of acupoints and, accordingly, there are large variations in the choice of acupoints among different acupuncturists [23, 24]. The application of data mining technology to the clinical research literature of ancient acupuncture and moxibustion has revealed that many studies have analyzed the selection of meridians and acupoints used by different practitioners. These studies provide evidence supporting the traditional theories of acupuncture treatment for a variety of diseases including knee osteoarthritis, diarrhea, migraine, vertigo, and lumbar disc herniation [25–29]. Based on a comprehensive set of quantitative clinical data, a recent article from our laboratory proposed that acupoints should be depicted according to the frequency of their use for the treatment of low back pain [8]. However, to date, there has been a lack of systematic research aimed at furthering the current understanding of the characteristics of the indications of each acupoint or attempting to visualize the meridian systems in terms of disease location. Thus, using data mining methods, the present study analyzed the characteristics of the indications of Source points and attempted to characterize the relationships among these acupoints and the different disease sites described in the classic Korean medical text* Chimgoogyeongheombang*.

## 2. Methods

### 2.1. Source of the Data

*Chimgoogyeongheombang (Experiential Prescriptions of Acupuncture and Moxibustion)* is a representative Korean medical text written by the royal physician Heo Im in 1644 and was the first book to specialize in acupuncture and moxibustion treatment during the Joseon dynasty [30, 31]. As we can see according to its own title, this text contains prescriptions for acupuncture and moxibustion that were obtained from transmitted medical texts and Heo Im's own clinical experiences. In his practice, Heo Im initially referred to authoritative medical texts such as* Huangdineijing (Huangdi's Internal Classic)*,* Qianjinfang (Prescriptions Worth a Thousand Gold)*,* Tongrenjing (Classic of the Bronze Figure)*,* Zhenjiuzishengjing (Classic of Nourishing Life with Acupuncture and Moxibustion)*,* ShenyingJing (Classic of Wondrous Response)*, and* Qixiaoliangfang (Formulae of Miraculous Effect)* from China and* Dongeuibogam (Treasured Mirror of Eastern Medicine)* from Korea. After accumulating a wide range of knowledge concerning acupuncture and moxibustion, however, he compiled his own medical theories and prescriptions. Heo Im's understanding of the causes of disease was usually based on meridian theory and internal organ theory [30] and he provided optimized descriptions of his own clinical experiences using his own language. In addition to providing his theories of the causes of disease, Heo Im also proposed corresponding prescriptions for each disease using acupuncture and moxibustion based on his clinical experience.

### 2.2. Database Construction

*Chimgoogyeongheombang* can be divided into two parts: general theory and clinical details. The clinical details section consists of 43 chapters that describe information regarding the acupoints that are associated with particular diseases or symptoms; 13 of these chapters are named according to disease sites (chapter of the head and face, chapter of the ears, chapter of the neck, etc.). All relevant information concerning the acupoints and disease sites used in the present study were extracted from the 13 chapters and categorized into the following 20 individual disease sites: head, face, ears, eyes, mouth, tongue, teeth, nose, throat, neck, chest, heart, hands, upper limbs, abdomen, flank, back, lower limbs, knees, and feet. A total of 471 acupoints were used to treat these 20 different disease sites and 110 acupoints remained after the removal of duplicates. As the Source point is the representative acupoint of the relationship between the spatial specificity of the meridian and the disease location, we analyzed the frequency of the cooccurrences of the Source points and disease sites among* Chimgoogyeongheombang*. Out of 12 Source points, 8 Source points were used to treat different disease sites. Of these 8 Source acupoints, none were related to the neck and the knee, and 18 disease sites were included in the final analyses.

### 2.3. Data Mining and Visualization of the Indications of Acupoints

Following construction of the database, data regarding the frequency of the co-occurrences of the 8 Source points and 18 disease sites were extracted to better understand the relationships among these factors. More specifically, the present study assessed which disease sites were meaningfully associated with a specific acupoint. To accomplish this, a term frequency-inverse document frequency (tf-idf) weighting scheme was applied to the cooccurrence table.

**Figure 1:**
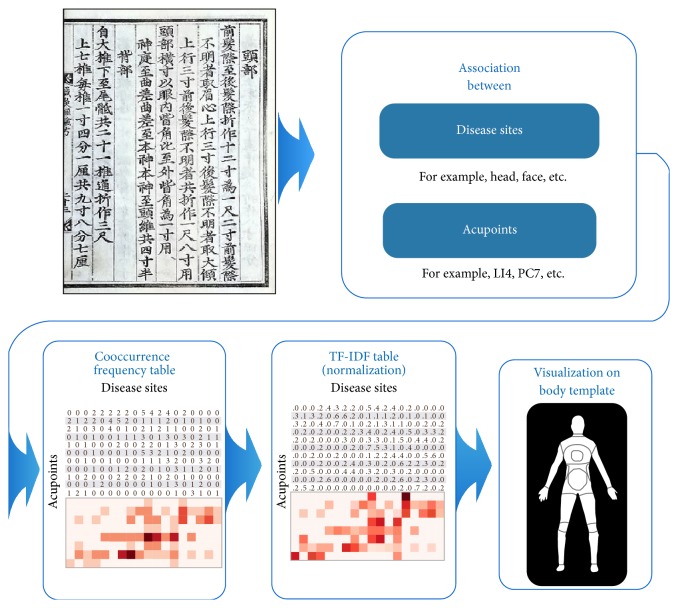
Procedures for data mining and the visualization of the indications of acupoints on the body map. The frequency of the cooccurrences of the 18 disease sites and the 8 Source points and the relationships among these factors provided valuable information regarding their clinical implications. To normalize the data, a term frequency-inverse document frequency (tf-idf) weighting scheme was applied to the cooccurrence table. The indications of each acupoint (tf-idf value) were visualized on a human body template.

The tf-idf method is one of the most widely used weighting schemes in the data mining research field, especially for information retrieval systems [31], because it quantifies the significance of particular terms in a document. Additionally, the present study quantified the significance of the associations between the disease sites and acupoints. In the tf-idf scheme, term frequency (tf_[*t* · *d*]_) refers to the number of times that term “*t*” occurs in document “*d*” and, therefore, tf_(*t*,*d*)_ represents how relevant term “*t*” is to document “*d*”. Document frequency (df_*t*_) is the number of documents that contain term “*t*” and, therefore, df_*t*_ represents the rarity of a term within the system of documents. Across the document system, rare terms are more informative than frequent terms and, thus, the inverse document frequency of “*t*” (idf_*t*_) is positively related to the informativeness of “*t*”. Arithmetically, idf is defined as log⁡(*N*/df_*t*_) instead of *N*/df_*t*_ to where *N* is the number of whole documents in order to diminish the effect of idf. Tf-idf_(*d*,*a*)_ is defined by assigning “disease site” to “term” and “acupoint” to “document” so that it quantifies the significance of the relationship of a specific disease site with a specific acupoint. Based on the tf-idf_(*d*,*a*)_ values, each acupoint is represented by a vector of tf-idf weights in an 18-dimension vector space (18 disease sites). Finally, the calculated tf-idf weights of each acupoint were normalized by the following cosine normalization: ($1/\sqrt{w_{1}^{2}+w_{2}^{2}+w_{3}^{2}\cdots +w_{M}^{2}}$). The relationships among the acupoints and disease sites were only described in the present study if they exhibited a tf-idf value greater than 0.4 (Figure 1).

The algorithm was intended to identify the disease sites that were related to the acupoints described in* Chimgoogyeongheombang*. All the tf-idf values were calculated using scikit-learn, which is a full-featured machine-learning package for the python programming language (http://scikit-learn.org/). The tf-idf_*(d,a)*_ values were overlaid on a human figure template using matplotlib, which is a plotting library for python (http://matplotlib.org/ [32]). A variety of relationships between a specific disease site and a specific acupoint were labeled according to the tf-idf_*(d,a)*_ values.

## 3. Results

### 3.1. Characteristics of the Indications of Acupoints

**Figure 2:**
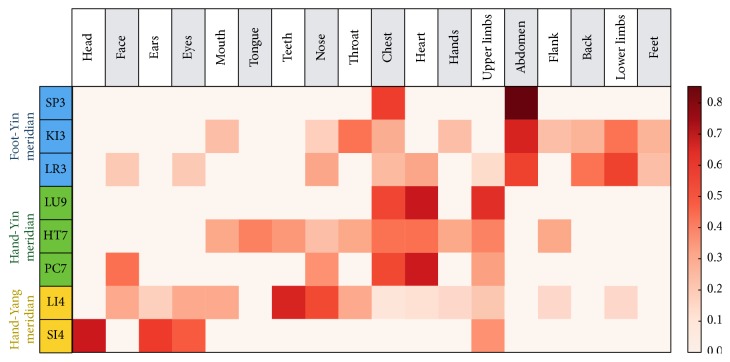
Characteristics of the indications of acupoints. The 8 Source points are presented on the *y*-axis of the array and the 18 selected disease sites are presented on the *x*-axis of the array. The 8 Source points were allocated with each meridian group (Foot-Yin, Hand-Yin, and Hand-Yang).

The 8 Source points are presented on the *y*-axis of the array in Figure 2. The 18 selected disease sites are presented on the *x*-axis of the array in Figure 2; the 8 Source points were highly associated with the 18 disease sites. Based on the Yin/Yang and Hand/Foot groupings, meridians can be categorized into the following three groups: Foot-Yin, Hand-Yin, and Hand-Yang.

In the Foot-Yin meridian, acupoint SP 3 showed superior tf-idf values with the abdomen (tf-idf: 0.85) and the chest (tf-idf: 0.53), acupoint KI 3 showed superior tf-idf values with the abdomen (tf-idf: 0.60), the lower limb (tf-idf: 0.40), and the throat (tf-idf: 0.40), and acupoint LR 3 showed superior tf-idf values with the abdomen (tf-idf: 0.52), the lower limb (tf-idf: 0.52), and the back (tf-idf: 0.40).

In the Hand-Yin meridian, acupoint LU 9 showed superior tf-idf values with the heart (tf-idf: 0.64), the upper limb (tf-idf: 0.57), and the chest (tf-idf: 0.51), acupoint HT 7 showed superior tf-idf values with the heart (tf-idf: 0.41) and the chest (tf-idf: 0.40), and acupoint PC 7 showed superior tf-idf values with the heart (tf-idf: 0.63), the chest (tf-idf: 0.50), and the face (tf-idf: 0.41).

In Hand-Yang meridian, acupoint LI 4 showed superior tf-idf values with the teeth (tf-idf: 0.60) and nose (tf-idf: 0.50) and acupoint SI 4 showed superior tf-idf values with the head (tf-idf: 0.64), the ears (tf-idf: 0.53), and the eyes (tf-idf: 0.46).

### 3.2. Visualization of the Indications of Acupoints on the Body Map

**Figure 3:**
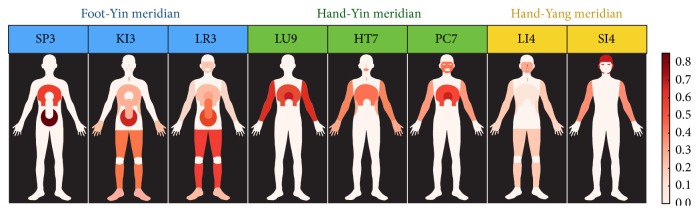
The visualization of the indications of the acupoints on a body map. Eight Source points representing the three meridian systems (Foot-Yin, Hand-Yin, and Hand-Yang meridian) and the indications of each acupoint (tf-idf value) were visualized on a human body template.

Eight Source points that represented each meridian system, as assessed using the tf-idf value, were visualized on a human body template. Thus, it was possible to visually display the spatial patterns of the various disease sites as they related to each acupoint (Figure 3).

The Source points on the left part (SP 3, KI 3, and LR 3), which are representative acupoints of the Foot-Yin meridian system, were highly associated with the truncus (including the abdomen and the chest) and the lower limbs. The Source points in the middle part (LU 9, HT 7, and PC 7), which are representative acupoints of the Hand-Yin meridian system, were highly associated with the upper truncus (including the heart and the chest) and upper limbs. The Source points in the right part (LI 4 and SI 4), which are representative acupoints of the Hand-Yang meridian system, were highly associated with the head regions (including the head, the face, the ears, the eyes, the mouth, the teeth, and the nose) and the upper limbs.

Additionally, there were similarities between the spatial patterns of the constellations of the indications of each acupoint and the routes of the corresponding meridians according to texts such as* Illustrations of Meridians and Collaterals in Ancient Times* and* Diagram of the Circulatory Course of Meridians*.

## 4. Discussion

The present study used data mining methods to analyze the characteristics of the indications of acupoints based on a classic text of Korean medicine,* Chimgoogyeongheombang*. Using the frequency of cooccurrences among 18 disease sites from* Chimgoogyeongheombang* and 8 Source points and the normalized relationships (tf-idf values) among these factors, the present study identified valuable data regarding the indications of each acupoint (Figure 2). For instance, the Source points of the Foot-Yin meridian, that is, SP 3, KI 3, and LR 3, were highly associated with the abdomen (tf-idf values: 0.85, 0.60, and 0.52, resp.). In contrast, the Source points of the Hand-Yin meridian, that is, LU 9, HT 7, and PC 7, were highly associated with diseases of the chest (tf-idf values: 0.51, 0.40, and 0.50, resp.) and heart (tf-idf values: 0.64, 0.41, and 0.63, resp.). Each of these relationships can be explained by the routes of the meridian systems. These findings suggest that the characteristics of the indications of each acupoint are primarily associated with their corresponding meridian system.

The present study also used tf-idf values to visualize the spatial patterns of the disease sites on the body as they related to each acupoint (Figure 3). For example, LI 4 in the Large Intestine meridian was highly associated with the constellations of the teeth and nose (tf-idf values: 0.60 and 0.50, resp.). The relationships between each of the acupoints and the constellations of the indications of the acupoints can be explained by the routes of the meridian systems. According to the classic text* Diagram of Meridians and Collaterals*, all three Hand-Yin meridians extend from the upper truncus, including the heart and chest, to the medial part of hands; all three Hand-Yang meridians extend from the lateral part of the hands to the head; all three Foot-Yang meridians extend from the head to the anterior and lateral parts of the foot; and all three Foot-Yin meridians extend from the medial part of the foot to the lower truncus. A comparison of the spatial patterns of the constellations of the indications of each acupoint with the routes of their corresponding meridians according to* Diagram of Meridians and Collaterals* reveals that the routes of the meridian systems likely demonstrate the constellations of the indications of the acupoints.

The present study has several limitations. First, the characteristics and spatial patterns of the indications of the acupoints were extracted from a single classic Korean medical text with limited bibliographical data. There are large variations from text to text when describing the relationships between disease sites and acupoints. Thus, it is necessary to further investigate the similarities and/or differences of the indications of acupoints using a variety of classic medical books from several cultures. Second, the present study assumed that the spatial patterns of the indications of each acupoint in terms of the 18 selected disease sites were highly associated with the routes of corresponding meridian systems but the available spatial data for the body were limited and did not include detailed spatial location information, such as the medial or lateral parts of the hand. However, based on the global distribution of the spatial patterns of the indications of each acupoint, it is possible to utilize the characteristics of the meridian systems as medical infographics. Third, the present findings were obtained based on the description of one individual's clinical observations from a classic medical textbook and not using outcomes extracted from well-structured clinical data. In order to fully characterize the characteristics of the indications of each acupoint to properly inform clinical assessments, it is necessary to investigate the relationships among the disease sites and acupoints using detailed spatial information from large-scale clinical investigations. Last but not least, when the Source points, that is, SP3 or LU 9 (*n* < 4), were not used frequently in the text, we cannot fully rule out the possibility of tf-idf values from the acupoints being exaggerated or distorted.

In conclusion, the present findings demonstrate that the indications of each acupoint are primarily associated with their corresponding meridian system. These findings also suggest that the routes of the meridian systems have clinical implications regarding the constellations of the indications of acupoints. The present authors strongly believe that the characteristics of the meridian system can act as an ancient infographic that will aid in the development and characterization of the clinical implications of acupoint selection for the treatment of various diseases.
